# Streptococcus constellatus bacteremia causing septic shock following tooth extraction: a case report

**DOI:** 10.1186/1757-1626-2-6493

**Published:** 2009-05-18

**Authors:** Kay Wei Ping Ng, Amartya Mukhopadhyay

**Affiliations:** Department of Medicine, National University Hospital5 Lower Kent Ridge RoadSingapore 119074

## Abstract

**Introduction:**

Organisms of the *Streptococcus milleri* group consists of *Streptococcus* *intermedius*, *Streptococcus constellatus*, and *Streptococcus anginosus*. Though they are commonly associated with abscesses, bacteremia with subsequent septic shock and mortality is infrequent.

**Case presentation:**

We report a case of a 27-year-old Chinese man who presented with *Streptococcus constellatus* bacteremia following an incomplete tooth extraction resulting in septic shock.

**Conclusion:**

Bacteremia from the *Streptococcus milleri* group is infrequent but must trigger the search for an underlying abscess. Knowledge of each member's predilection for different clinical presentations can aid in determining the location of the underlying source of infection.

## Introduction

Organisms of the *Streptococcus milleri* group consists of *Streptococcus* *intermedius*, *Streptococcus constellatus*, and *Streptococcus anginosus*. Though they are commonly associated with abscesses, bacteremia with subsequent septic shock and mortality is infrequently described in the literature. We report a case with such a presentation. Our case also illustrates how each organism of the *Streptococcus milleri* group has its own commonly associated clinical manifestations, and how this knowledge is necessary in the search for its source and subsequent treatment.

## Case presentation

A 27-year-old Chinese man presented to the Emergency Department (ED) after collapsing outside a dental clinic. No further history was available at presentation. He had a Glasgow Coma Scale (GCS) score of 4 (E1V2M1), was febrile (41°C) and hypotensive (72/25 mmHg). He was given 3.5L of normal saline intravenously, and started on intravenous (IV) Dopamine. An arterial blood gas measurement on non-rebreather mask showed pH 7.24, PaCO_2_ 39 mmHg (35-45), PaO_2_ 374 mmHg (75-100), Bicarbonate 16mmol (23-33) and base excess −11 (−2−+2). He also developed generalised tonic-clonic seizures that was aborted with IV lorazepam. Due to his low GCS and severe metabolic acidosis, he was intubated and admitted to MICU.

Initial blood tests showed a white blood cell count of 11.80 × 10^9^(3.3-9.6), which was predominantly neutrophilic, Haemoglobin 13.6gm/dl (12.9-16.7) and platelet 168 × 10^9^ (162-427). Lactate was elevated at 3.6mmol/l (0.7-2.1). Serum electrolytes and Urea were normal but Creatinine was elevated at 142µmol/L (65-125).

He had disseminated intravascular coagulopathy (DIVC) with an INR of 3.54, PT 36.0sec (12-14.8), PTT 121.4 sec (28.4-39.7), and fibrinogen 0.66 g/L. Platelets dropped from 168 × 10^9^ to 28 × 10^9^ within hours of admission, and he required cryoprecipitate and platelet transfusions. He had an elevated AST of 143U/L (10-50) and LDH 3180 U/L (300-700), consistent with severe sepsis. He was thus in multi-organ dysfunction on admission. However, he had adequate urine output and did not require any renal replacement therapy.

Given his abrupt neurologic impairment with multi-organ failure, initial differential diagnoses included thrombotic thrombocytopenic purpura, gram negative sepsis, leptospirosis and meningo-encephalitis. Initial antibiotic cover was IV ceftriaxone and IV acyclovir for possible meningo-encephalitis, and IV metronidazole was added to cover for hepatobiliary sepsis in view of the deranged liver function tests.

## Progress

Computed Tomography (CT) scan of the head showed no intracranial abnormalities and a detailed toxicology screen was negative. Lumbar puncture was traumatic but was negative for bacteria, acid fast bacilli, and fungus.

His Procalcitonin was 19.77ug/L (<0.5), suggestive of bacterial infection. Malaria blood film, dengue and leptospiral serology were negative, as were Chikungunya virus, Hanta virus total Ab-IF, *B.* *pseudomallei* antibodies, stool for enterovirus and coxsackie virus, and Human Immunodeficiency Virus screen. Stool cultures were negative for *E.* *coli* 0157:H7. CT scan of the abdomen and pelvis showed no focus of infection.

A peripheral blood film showed a left shift, with schistiocytes, but no significant spherocytosis or polychromasia, consistent with DIVC.

By this time, our patient's room-mate was contacted. Further history from him revealed that our patient had seen the dentist for a toothache. The dentist reported that the patient had undergone a partial extraction of his right first lower molar tooth, and pus was noted in the socket. Immediately after, he developed chills and rigors but left the dental clinic. He was subsequently found collapsed outside the clinic. He had no other significant past medical or travel history.

In view of the new history, CT scan of the neck was requested. This showed a focal area of bony erosion at the right side of the mandible associated with the right first molar tooth. No adjacent abscess collection or venous thrombosis was seen. He was reviewed by the oral-maxillary surgeon who found no local abscess requiring urgent drainage.

Our patient continued to be obtunded even when his sedation was stopped. A Magnetic Resonance Imaging of the head showed several small acute infarcts in the right parietal white matter, genu of the corpus callosum and the right thalamus, consistent with septic emboli. There was no thrombosis in the cavernous or cerebral venous sinuses. An Electroencephalogram (EEG) did not show epileptiform activity.

A transthoracic echocardiogram showed a patent foramen ovale. There was an anterior leaflet mitral valve prolapse with mild mitral valve regurgitation, but no visible vegetations.

Four days after admission, his blood culture taken on admission grew *Streptococcus constellatus*, sensitive to penicillin, ampicillin, erythromycin and clindamycin.

His diagnosis was *S.* *constellatus* septic shock secondary to a right periodontal abscess, with septic emboli to the brain via the patent foramen ovale. Acyclovir and Metronidazole was stopped, and he was converted to IV crystalline penicillin 4 MU every 4 hours. He improved clinically, was extubated on the sixth day following his admission and discharged to the general ward.

He subsequently underwent an extraction of the lower right wisdom tooth and root fragments of the lower right first molar. A cystic cavity was found underneath the root fragment. The removed cystic tissues were culture negative for bacteria.

He underwent aggressive rehabilitation. He completed 4 weeks of intravenous penicillin therapy and was given oral amoxicillin 500 mg 3 times a day for a further 3 weeks. He has since resumed his usual lifestyle with only a mild residual dysarthria.

## Discussion

*Streptococcus constellatus* is a member of the *Streptococcus milleri* group which includes the three species, *S. intermedius, S.anginosus* and* S. constellatus*. These organisms are often α-haemolytic, but occasionally can be β-haemolytic or non-haemolytic [1]. They are found among normal oropharyngeal and gastrointestinal flora, but can cause abscesses in the abdominal cavity, lower respiratory tract, urogenital tract, orofacial and sinus area and skin. They can spread hematogenously to cause metastatic abscesses in the brain, liver, spleen, subdural space, bone, as well as endocarditis [1-4]. *S. milleri* has a predisposition to form abscesses [1-3,5], but the exact reason is not clear. Possibilities include the organisms' polysaccharide capsule and synergistic activity with anaerobes [2]. Conversely, bacteremia with this group of organisms is infrequent [5].

Due to the close phenotypic analysis of *S. milleri,* and the difficulty with identifying the three isolates [1], correlation with clinical syndromes has not been consistent. However, it has been noted that the different species of the group have associations with different clinical syndromes [4]. Generally, *S. intermedius* and* S. constellatus* are more associated with abscesses than *S. anginosus*. *S. intermedius* also appears to cause more deep-seated abscesses than those due to *S.* *constellatus,* which are more often superficial [4]. *S.* *constellatus* also appears to cause a broader range of infections, including odontogenic disease, but does not appear to predominate in any particular site [2,4], with the possible exception of increased prevalence in abdominal specimens [3,4] and the respiratory tract [2]. Abscesses caused by *S.* *constellatus* were more likely to be polymicrobial, with the co-isolate reflecting the site from which the specimen was obtained [4].

*S. anginosus* is the most frequent species in bacteremia [4,6], while *S. constellatus* is detected only infrequently in the blood [4]. Bacteremic patients may have an underlying comorbidity, including hepatic and biliary disease, neoplasia, and diabetes [5,6]. While other viridans streptococci commonly causes bacteremia in neutropenic patients [7], the *S. milleri* group seldom does [6]. *S. milleri* bacteremia is often associated with a suppurative foci of infection [5,6,8], most commonly from an intra-abdominal source of sepsis, including hepatobiliary sepsis such as cholangitis or cholecystitis without abscess formation [8,9], and from a bronchopulmonary source [6]. It can also result from a disruption of the mucosal digestive barrier acting as a portal of entry [6]. Multiple positive blood cultures and polymicrobial cultures among patients are especially suggestive of a suppurative focus of infection [6].

In a series by Frédéric Bert et al. [6], signs of shock were present in only 4 out of the 51 patients with *S. milleri* bacteremia, with only 2 deaths out of these 51 patients. The mortality rates of bacteremic patients have been variable, and has been quoted as 12.5% in one series by Casariego et al [5], to as high as 26% in another series by Jacobs et al, though this was mainly among severely ill patients [8].

In view of the cerebral emboli, infective endocarditis was considered, although the cerebral emboli could have resulted via seeding through the patent foramen ovale. Infective endocarditis has been found to be associated with a significant proportion of patients with *S. milleri* bacteremia [5], although it is more common in viridans streptococci bacteremia [9]. A study of infective endocarditis caused by *S.* *milleri* group organisms found that 25% of the cases had extracardiac suppurative foci of infection, 14% had intracardiac abscesses, and 90% had valvular regurgitation [10].

*S. milleri* organisms are generally susceptible to penicillin, ampicillin and ceftriaxone. Variable susceptibility to tetracycline, clindamycin, and erythromycin has been reported [4,11]. Bantar et al. [3] studied the susceptibilities of each strain to penicillin, erythromycin, vancoycin, gentamicin and streptomycin, and found decreased susceptibility in 12.5% of *S. anginosus*, 5.5% of *S. constellatus*, and 33.3% of *S*. *intermedius*. Although there are significant differences in susceptibility to beta-lactam antibiotics among the different *S.* *milleri* species, the differences are sufficiently small to be clinically irrelevant. Species identification therefore may not help to predict antibiotic susceptibility and aid in choice of empiric therapy [11]. Clarridge et al also found that all members of the *S. milleri* group were susceptible to levels of penicillin, amoxicillin, cefotaxime or ceftriaxone achieved by the usual dosage [4]. However, as the source of these organisms are often abscesses, the best treatment would include detection and surgical drainage.

## Conclusion

Our patient illustrates the unusual presentation of severe septic shock from *Streptococcus constellatus* bacteremia. The knowledge of the different clinical associations of *S. intermedius, S. constellatus,* and *S.* *anginosus* also aid in the management of the patient in directing the search for an associated infection or occult abscess.
